# Supplementary figures and images for: A comprehensive atlas of white matter tracts in the chimpanzee
Source: PLoS Biol. 2020 Dec 31;18(12):e3000971. doi: 10.1371/journal.pbio.3000971 (PMC7806129; doi:10.1371/journal.pbio.3000971)

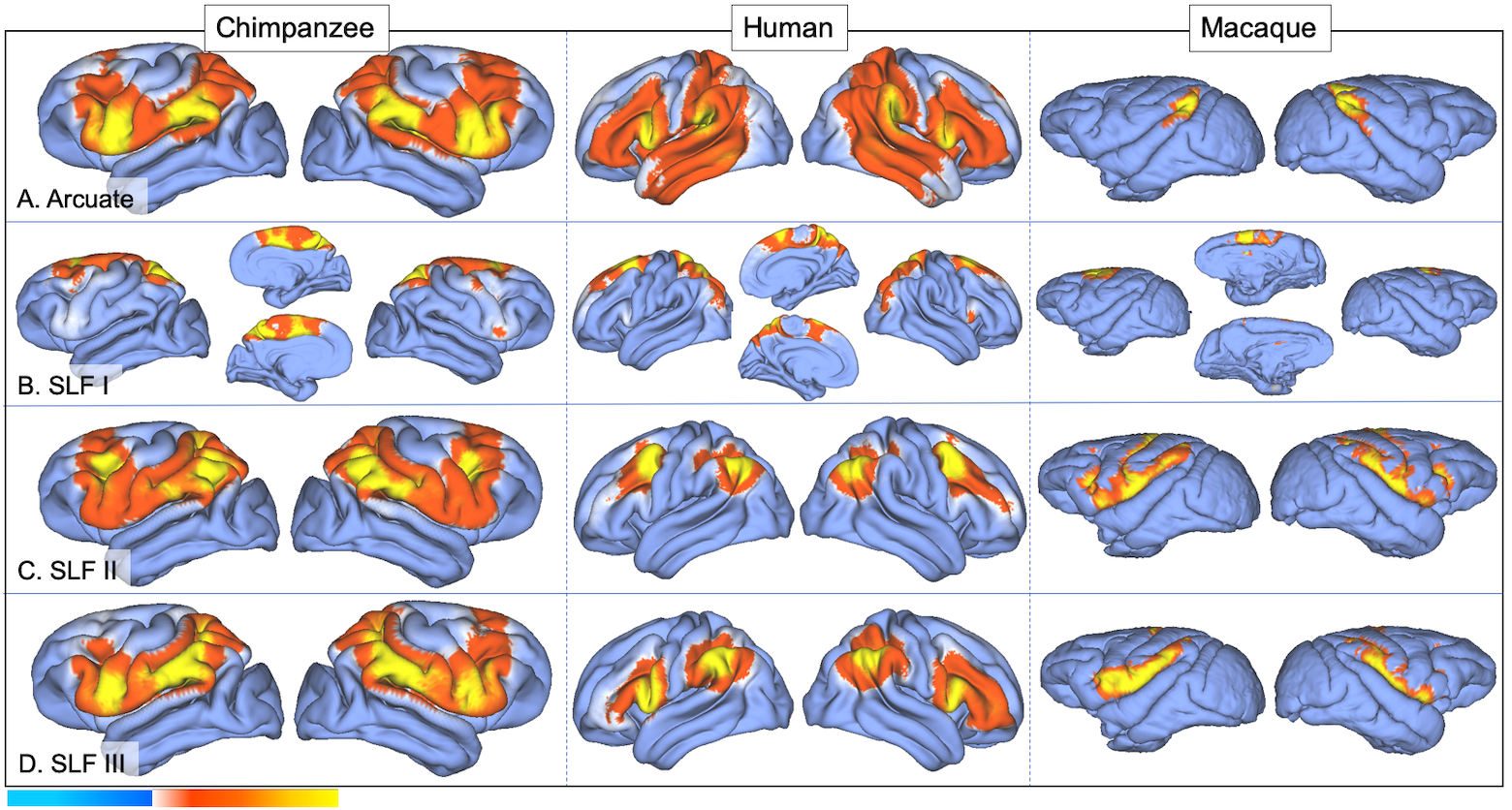

Supplement: S1 Fig — Surface projection results for arcuate (A) and SLFs I-III (B–D) in chimpanzee, human, and rhesus macaque. Color bar indicates heat map of tractogram normalized probability values. Surface projections are available at https://git.fmrib.ox.ac.uk/rmars/chimpanzee-tractography-protocols/-/tree/master/surface_projections. (TIFF) [file pbio.3000971.s001.tiff]

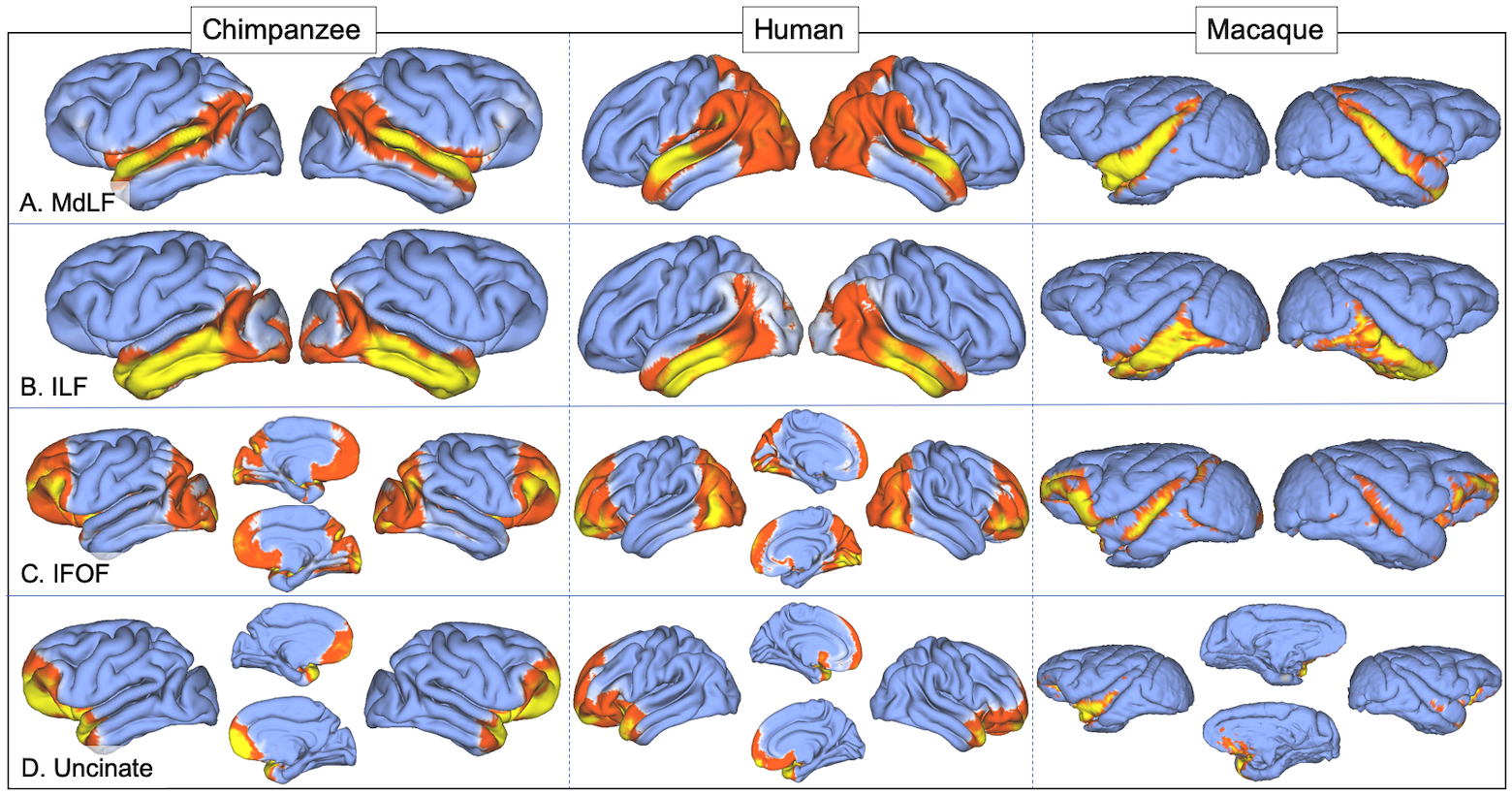

Supplement: S2 Fig — Surface projection results for MdLF (A), ILF (B), IFOF (C), and UF (D) in chimpanzee, human, and rhesus macaque. Surface projections are available at https://git.fmrib.ox.ac.uk/rmars/chimpanzee-tractography-protocols/-/tree/master/surface_projections. (TIFF) [file pbio.3000971.s002.tiff]

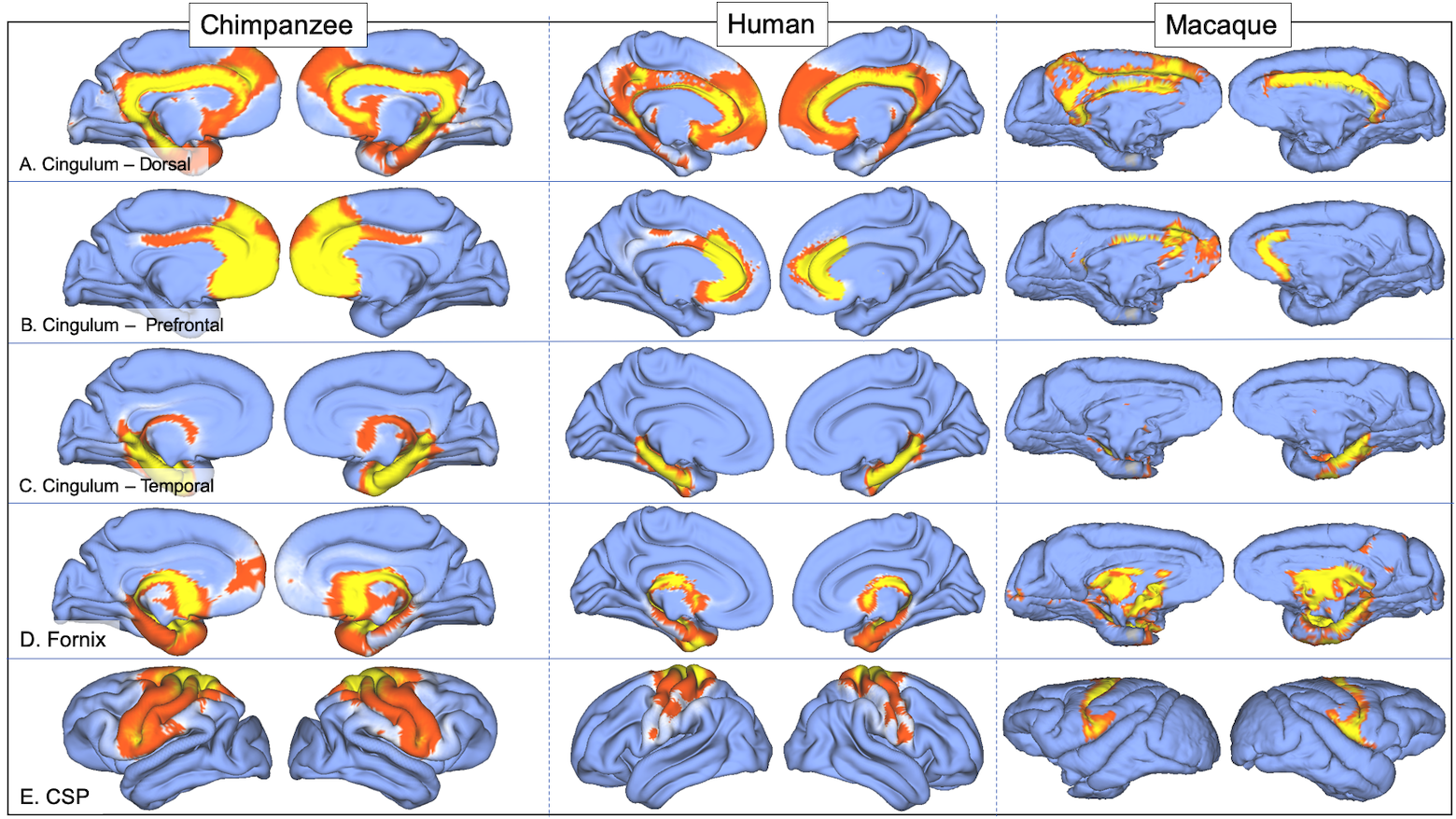

Supplement: S3 Fig — Surface projection results for cingulum bundle (A–C), fornix (D), and CST (E) in chimpanzee, human, and rhesus macaque. Surface projections are available at https://git.fmrib.ox.ac.uk/rmars/chimpanzee-tractography-protocols/-/tree/master/surface_projections. (TIFF) [file pbio.3000971.s003.tiff]

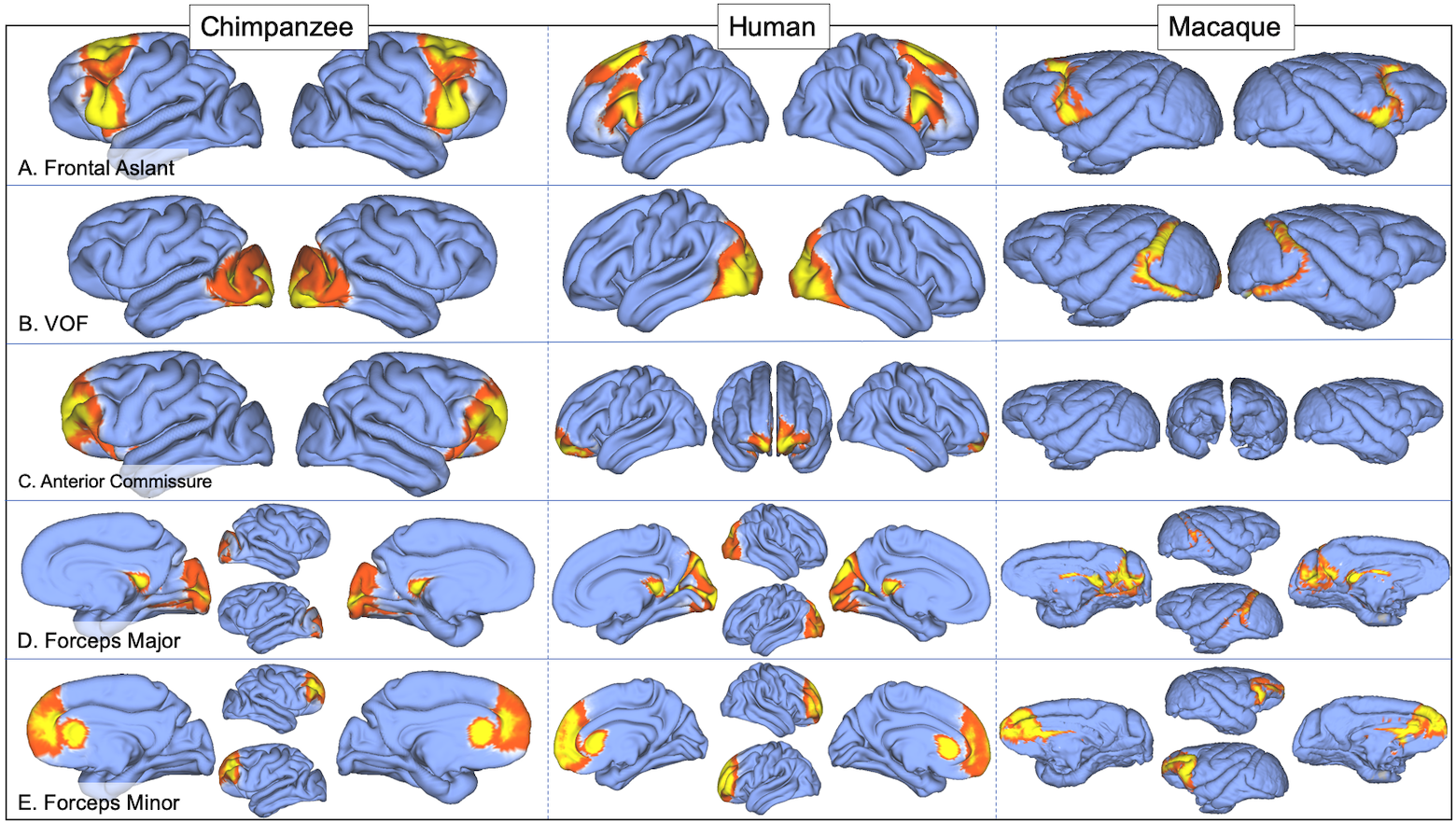

Supplement: S4 Fig — Surface projection results for frontal aslant (A), VOF (B), anterior commissure (C), and forceps major and minor (D, E) in chimpanzee, human, and rhesus macaque. Surface projections are available at https://git.fmrib.ox.ac.uk/rmars/chimpanzee-tractography-protocols/-/tree/master/surface_projections. (TIFF) [file pbio.3000971.s004.tiff]

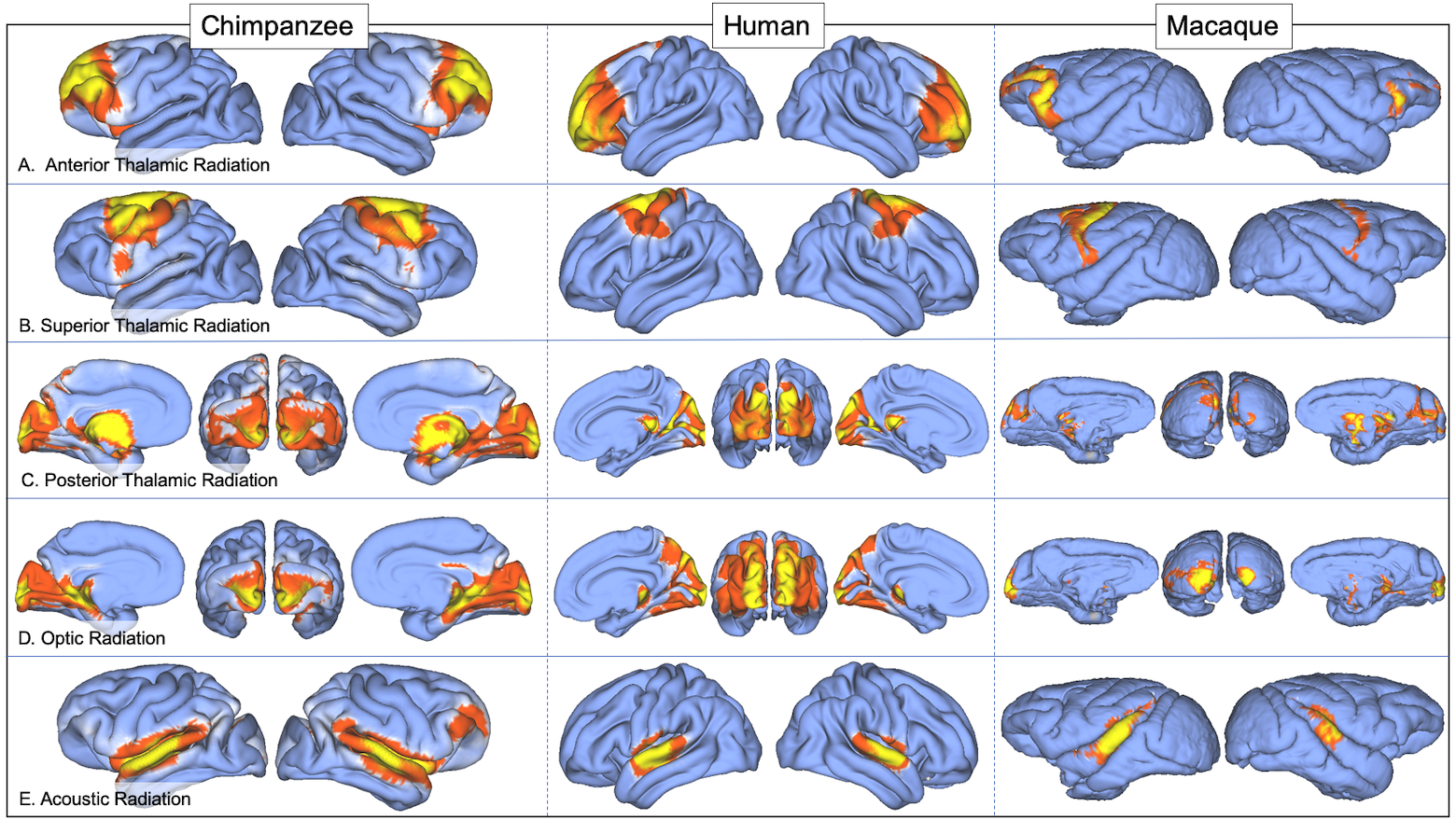

Supplement: S5 Fig — Surface projection results for anterior, superior, and posterior thalamic radiations (A–C), optic radiation (D), and acoustic radiation (E) in chimpanzee, human, and rhesus macaque. Surface projections are available at https://git.fmrib.ox.ac.uk/rmars/chimpanzee-tractography-protocols/-/tree/master/surface_projections. (TIFF) [file pbio.3000971.s005.tiff]

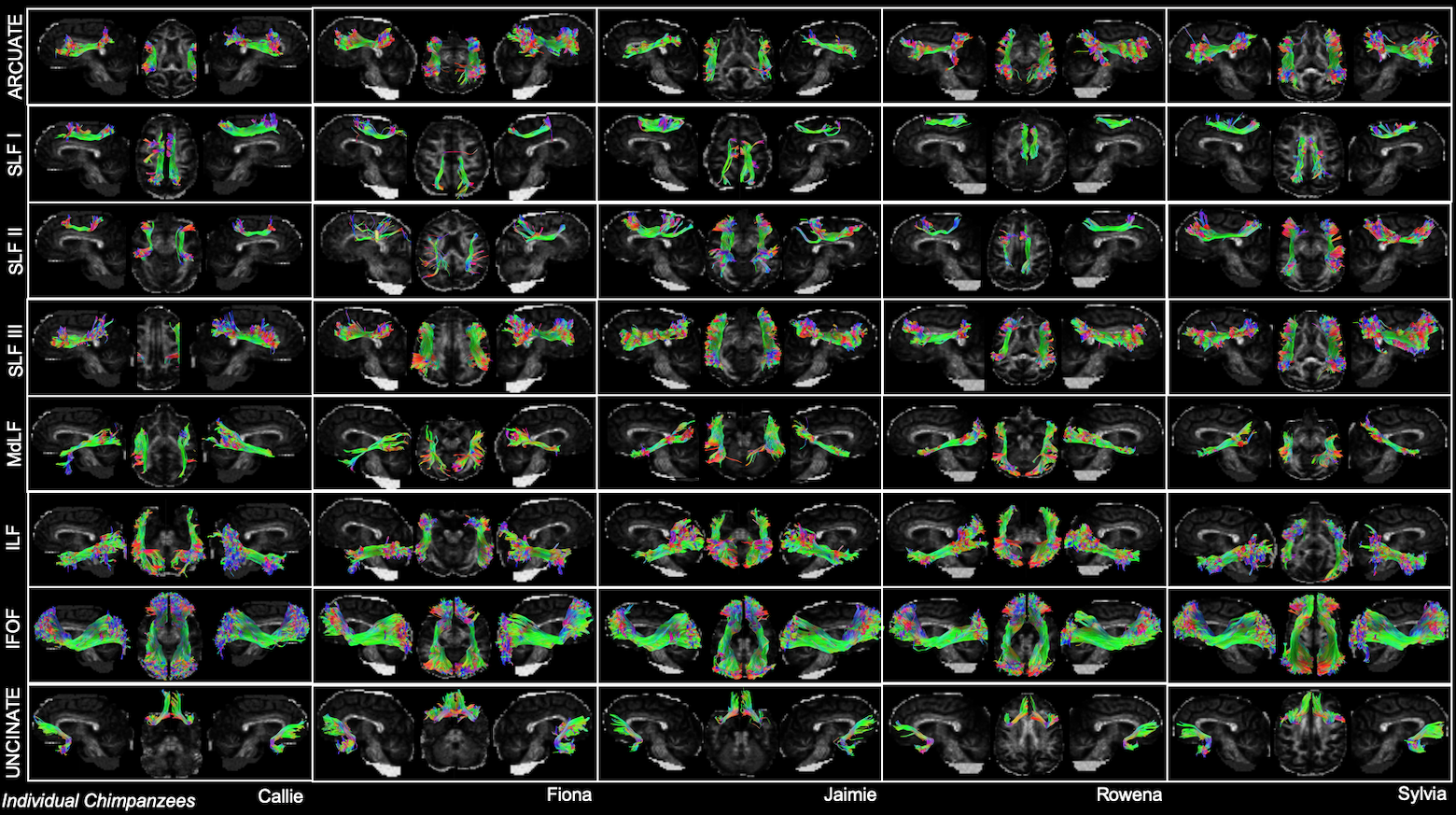

Supplement: S6 Fig — (TIFF) [file pbio.3000971.s006.tiff]
